# Association of Aggresomes with Survival Outcomes in Pediatric Medulloblastoma

**DOI:** 10.1038/s41598-019-49027-x

**Published:** 2019-08-30

**Authors:** Maha Yehia, Hala Taha, Asmaa Salama, Nada Amer, Amal Mosaab, Omneya Hassanain, Amal Refaat, Dina Yassin, Ahmed El-Hemaly, Soha Ahmed, Mohamed El-Beltagy, Osama Shaalan, Shahenda El-Naggar

**Affiliations:** 1 Department of Pathology, Children’s Cancer Hospital Egypt 57357, Cairo, Egypt; 2 Tumor Biology Research Program, Basic Research Unit, Department of Research, Children’s Cancer Hospital Egypt 57357, Cairo, Egypt; 3 Clinical Research Unit, Department of Research, Children’s Cancer Hospital Egypt 57357, Cairo, Egypt; 4 Department of Radiology, Children’s Cancer Hospital Egypt 57357, Cairo, Egypt; 5 Laboratory of Molecular Biology, Department of Clinical Pathology, Children’s Cancer Hospital Egypt 57357, Cairo, Egypt; 6 Department of Pediatric Oncology, Children’s Cancer Hospital Egypt 57357, Cairo, Egypt; 7 Department of Radiotherapy, Children’s Cancer Hospital Egypt 57357, Cairo, Egypt; 8 Department of Neurosurgery, Children’s Cancer Hospital Egypt 57357, Cairo, Egypt; 9 Department of Pathology, National Cancer Institute, Cairo University, Cairo, Egypt; 10 Department of Radiology, National Cancer Institute, Cairo University, Cairo, Egypt; 11 Department of Clinical Pathology, National Cancer Institute, Cairo University, Cairo, Egypt; 12 Department of Pediatric Oncology, National Cancer Institute, Cairo University, Cairo, Egypt; 13 Department of Clinical Oncology, Faculty of Medicine, Aswan University, Aswan, Egypt; 14 Department of Neurosurgery, Faculty of Medicine, Cairo University, Cairo, Egypt; 15 Department of Molecular Diagnostics, Genetic Engineering and Biotechnology Research Institute, University of Sadat City, Menoufia, Egypt

**Keywords:** CNS cancer, Protein folding

## Abstract

Aggresomes are inclusion bodies for misfolded/aggregated proteins. Despite the role of misfolded/aggregated proteins in neurological disorders, their role in cancer pathogenesis is poorly defined. In the current study we aimed to investigate whether aggresomes-positivity could be used to improve the disease subclassification and prognosis prediction of pediatric medulloblastoma. Ninety three pediatric medulloblastoma tumor samples were retrospectively stratified into three molecular subgroups; WNT, SHH and non-WNT/non-SHH, using immunohistochemistry and Multiplex Ligation Probe Amplification. Formation of aggresomes were detected using immunohistochemistry. Overall survival (OS) and event-free survival (EFS) were determined according to risk stratification criteria. Multivariate Cox regression analyses were carried out to exclude confounders. Aggresomes formation was detected in 63.4% (n = 59/93) of samples. Aggresomes were non-randomly distributed among different molecular subgroups (*P* = 0.00002). Multivariate Cox model identified aggresomes’ percentage at ≥20% to be significantly correlated with patient outcome in both OS (HR = 3.419; 95% CI, 1.30–8.93; *P* = 0.01) and EFS (HR = 3; 95% CI, 1.19–7.53; *P* = 0.02). The presence of aggresomes in ≥20% of the tumor identified poor responders in standard risk patients; OS (*P* = 0.02) and EFS (*P* = 0.06), and significantly correlated with poor outcome in non-WNT/non-SHH molecular subgroup; OS (*P* = 0.0002) and EFS (*P* = 0.0004).

## Introduction

Maintaining the integrity of the proteome is essential for cell viability^1,2^. Cells have developed protein quality control (PQC) machinery to monitor and maintain the delicate balance between protein synthesis, folding, and degradation^1,2^. The disruption of this balance by aging, mutation or cellular insults, leads to the accumulation of misfolded/aggregated proteins^3–5^. To avoid the consequent onset of proteotoxicity, misfolded proteins are targeted to the proteasome for degradation^6,7^. When protein aggregates exceed proteasome degradation capacity, they are shuttled by histone deacetylase 6 (HDAC6) and dynein motor proteins along the microtubules to microtubule-organizing center (MTOC)^8,9^. Protein aggregates are then deposited into cytoplasmic inclusion bodies termed aggresomes^10–12^. Aggresomes exhibit a structural organization containing misfolded proteins enclosed in a cage formed by vimentin intermediate filament^13,14^. Aggresome formation is recognized as a cytoprotective response to sequester potentially cytotoxic misfolded/unfolded proteins and facilitate their clearance by autophagy^15^. Accumulation of misfolded proteins is a pathological feature common to many neurodegenerative diseases, including Parkinson’s disease, Alzheimer’s disease, Huntington’s disease, and amyotrophic lateral sclerosis^15,16^. In cancer, aggresome formation is limited to certain tumor types, and their relationship to cancer pathogenesis is yet to be elucidated^14,17,18^. We have recently identified aggresomes in choroid plexus carcinoma (CPC) tumors^19^. The detection of aggresomes in CPC rather than the benign variant; choroid plexus papilloma (CPP) suggested that is a feature associated with the tumor aggressiveness^19^. Therefore, we decided to examine their presence and assess their prognostic value in other pediatric brain tumors primarily aggressive subtypes. One of the most common pediatric malignant brain tumors is medulloblastoma (MB)^20,21^. MB encompasses 3 main histological variants; classic, desmoplastic nodular/desmoplastic with extensive nodularity (D/N) and large cell/anaplastic (LC/A)^22^. Historically, risk stratification relied on clinicopathological variables including age, presence of metastases, extent of resection and histological subtypes^23,24^. Accordingly, MB patients at the age of 3 years and above are assigned to one of two risk groups according to Chang’s staging system, namely; standard risk (SRMB) (classic or desmoplastic histology, non-metastatic and total or near-total resection with a residual tumor less than 1.5 cm), or high-risk (HRMB) (large cell/anaplastic, classic with focal anaplasia histology, metastatic or subtotal resection >1.5 cm)^25^. MB patients at an age of less than 3 years old are assigned to an infant medulloblastoma (InfMB) risk group^26^. In recent years, extensive genomic studies of MB tumors identified 4 molecular groups termed Wingless (WNT), Sonic Hedgehog (SHH), Group 3 and Group 4^27–29^. While specific pathways are altered in both WNT and SHH MBs, Group 3 and Group 4 gene expression signatures are still somewhat ambiguous and may be combined into “non-WNT/non-SHH” subgroup^26,30^. These molecular subgroups differ in the cell of origin, genetic mutations, epigenetic and phenotypic profiles^27,30,31^. The combined use of both clinical and molecular classification provides superior risk stratification, and subsequently impacts treatment options^30^. Despite intensive molecular characterization of MB tumors, many patients progress or relapse on current treatment strategies, especially patients stratified as non-WNT/non-SHH^30,31^. In the current study we describe the characterization of aggresomes in pediatric MB tumors. We report that aggresomes are independent prognostic factor. Hence we propose that screening for aggresomes in pediatric MB tumor samples can be integrated with molecular stratification to establish a better model for predicting patients’ outcome.

## Results

### Patients’ characteristics

Immunohistochemistry (IHC) analysis was used to stratify MB tissue samples into three molecular subgroups; WNT, SHH or non-WNT/non-SHH (Supplementary Fig. 1A, Supplementary Table 1). Multiplex Ligation Probe Amplification Dosage Quotient (MLPA DQ) was used to identify tumors with monosomy 6 and thus further confirm the WNT molecular subgroup (Supplementary Fig. 1B). Clinical characteristics of patients based on molecular stratification are listed in (Table 1). For clinicopathological criteria, all 93 patients were included in the analysis. Reference histology, age at diagnosis, and metastatic stage were found to be non-randomly distributed among the molecular subgroups (*p-values* of 0.0001, 0.001, and 0.001, respectively).

**Table 1:** Clinicopathological criteria based on molecular subgroups.

	WNT (*n* = 8)	SHH (*n* = 23)	Non-WNT/Non-SHH (*n* = 62)	*P*-value
**Histologic** **subtypes**				0.0001*
Classic	5 (62.5%)	7 (30.43%)	38 (61.29%)	
D/N	0	10 (43.4%)	2 (3.22%)	
LC/A	3 (37.5%)	6 (26%)	22 (35.48%)	
**Age at diagnosis**				0.001*
<3	0	7 (30.4%)	3 (4.83%)	
≥3	8 (100%)	16 (69.5%)	59 (95.16%)	
**Stage** (n = 85)				0.005*
M0	7 (87.5%)	16 (69.56%)	29 (46.7%)	
M+	1 (12.5%)	3 (13%)	30 (48.3%)	
N/A	0	4 (17.3%)	3 (4.8%)	
**Resection**				0.5
R0	8 (100%)	18 (78.2%)	50 (80.6%)	
R+	0	5 (21.7%)	12 (19.3%)	
**Risk stratification** (n = 90)				0.005*
SRMB	4 (50%)	8 (34.78%)	15 (25.4%)	
InfMB	0	6 (26.08%)	1 (1.69%)	
HRMB	4 (50%)	9 (39.13%)	43 (72.88%)	

For statistical analysis correlation of metastatic stage, patients with unknown status were excluded, Statistical analysis was performed using Fisher Exact test. Abbreviations: NA = data not available. M0 = non-metastatic disease. M+= metastatic disease. R0 = gross total resection (<1.5 cm). R+= subtotal resection (≥1.5 cm).

### Characterization of aggresomes in MB

**Figure 1:**
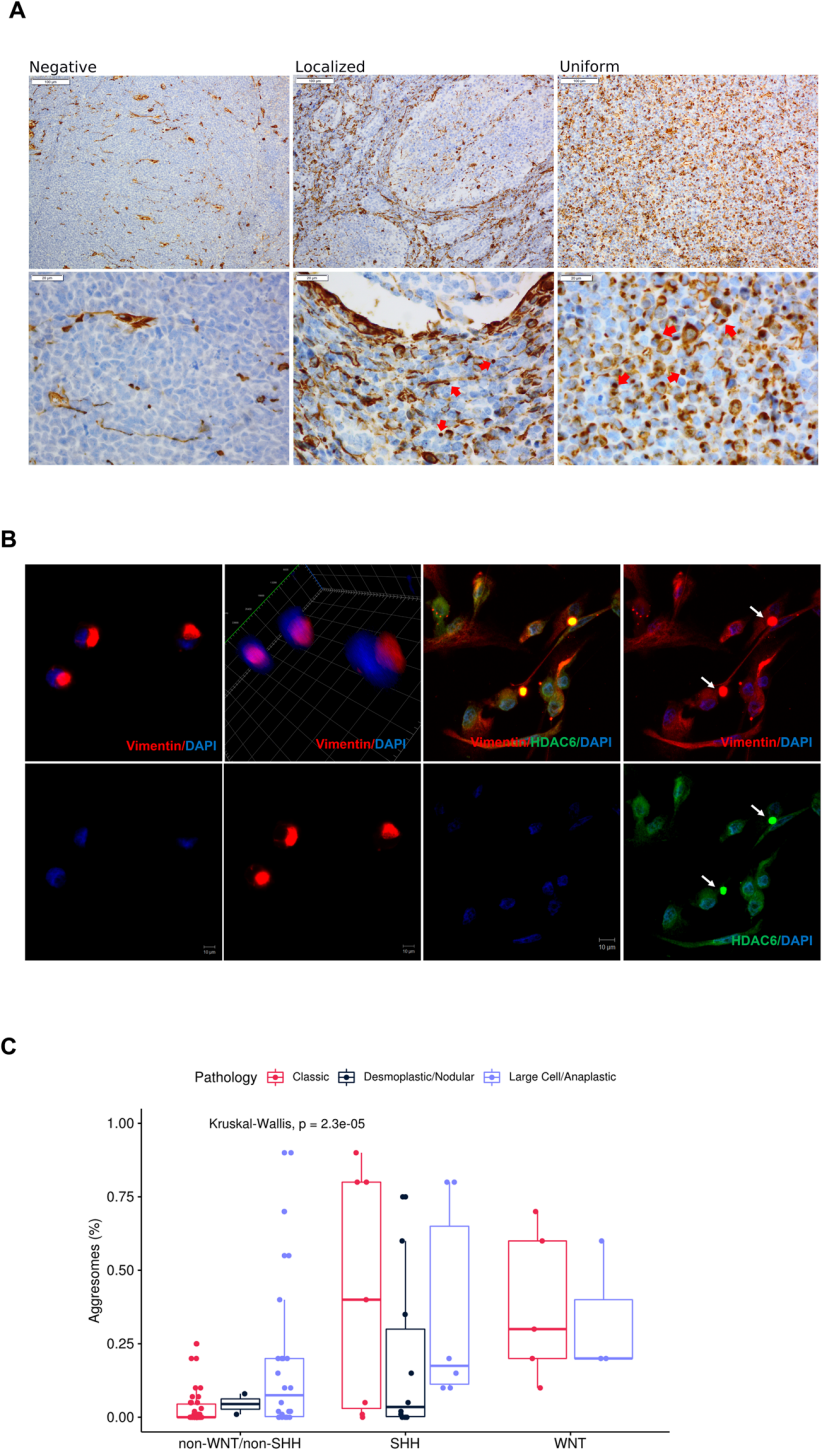
Characterization of aggresomes in pediatric medulloblastoma tumor samples. (**A**) IHC analysis of vimentin identified three different patterns. Negative; vimentin immunoreactivity was absent or only observed in infiltrating blood vessels. Localized; observed in desmoplastic/nodular variant, where juxta-nuclear dot-like vimentin was identified in the interstitial space between nodules. Uniform distribution; of juxta-nuclear dot-like were identified in classic and LC/A histological subtypes. Red arrows show para nuclear localization of vimentin indicative of aggresomes formation. (**B**) IF analysis of CCHE-188 cells. Cells were immunostained with mouse anti-vimentin and rabbit anti-HDAC6 then visualized using Alexa Fluor 488 goat anti-mouse and Alexa Fluor 555 goat anti-rabbit respectively. Cells were counter-stained using DAPI. (**C**) Bar Plot of the distribution of aggresomes percentages among different molecular subgroups, subdivided according to histological variant. *p-values* were calculated using Kruskal-Wallis test.

To identify aggresomes in tumor samples, vimentin immunoreactivity was tested in all cases. Only vimentin juxta nuclear dot was considered for aggresomes-positivity (59/93) representing 63.4% of tumor samples (Fig. 1A, Supplementary Table 1). For aggresome-positive tumors, aggresomes were either localized to interstitial cells between nodules as seen in D/N tumors, or uniform throughout all tissue section as seen in classic and LC/A tumors (Fig. 1A). CCHE-188 cell line was developed from an aggresomes-positive MB tumor sample in which aggresomes were detected in 25% of cultured cells (Fig. 1B). Cell pellet from CCHE-188 cells was compared to the original tumor and was found to have similar percentage of aggresomes, hence confirming that they are inherent structures of aggresome-positive tumor cells (Supplementary Fig. 2). Additionally, HDAC6 colocalized with vimentin only in aggresomes-positive cells (Fig. 1B). Upon examination of aggresome percentage in the tumor samples, it was found to be correlated with molecular subgroups (*P* = 0.00002) but not with histological variants (Fig. 1C).

### Association of aggresomes with clinical outcome of MB patients

**Figure 2:**
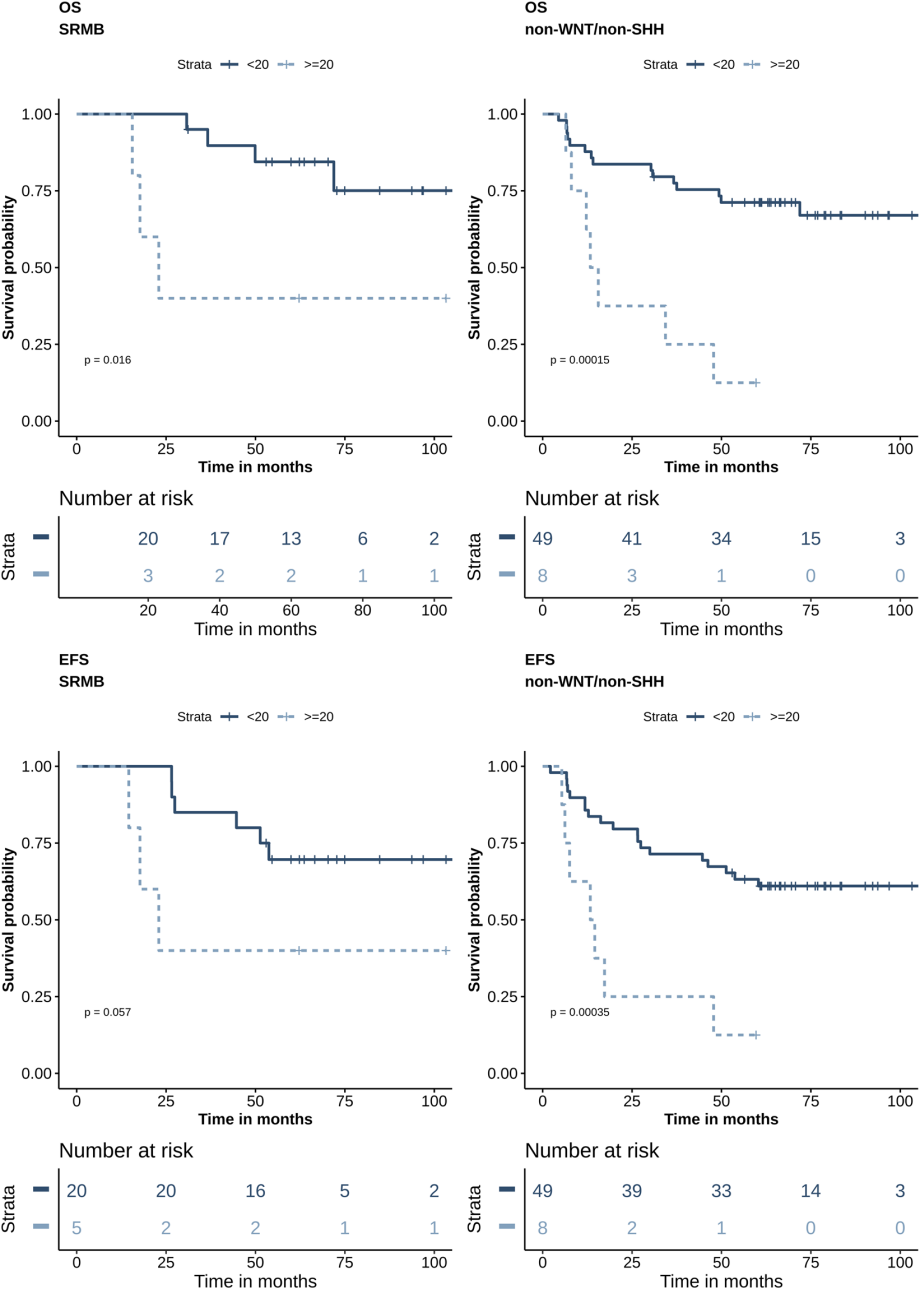
Aggresomes predict poor outcome among standard risk and non-WNT/non-SHH patients. Survival analysis Kaplan-Meier according to percentage of aggresomes within the tumor cells. (**A**) Patients are stratified according to clinicopathological risk criteria. (**B**) Patients are stratified using molecular subgrouping. Survival probability (y axis) and time indicated in months (x axis). *p-values* were calculated using the log-rank test.

To perform the survival based cut-off determination, aggresomes’ percentage was dichotomized into 5%, 10%, 20%, 30% and 60% and both time dependent area under the curve (AUC) and C-index were calculated (Supplementary Table 2). Accordingly, the 20% cut-off exhibited the best discriminatory value, hence subsequent analysis was performed using this cut-off. Univariate Cox proportional hazards analysis was performed with all risk factors and ≥20% of aggresomes (Table 2). Histological variants; OS (*P* = 0.007) and EFS (*P* = 0.04), molecular subgroups; OS (*P* = 0.08) and EFS (*P* = 0.05), and aggresomes’ percentage ≥20%; OS (*P* = 0.06) and EFS (*P* = 0.2), were found to have the highest significant impact on prognosis (Supplementary Fig. 3). Multivariate Cox model was then performed to identify relationships between risk factors and prognosis (Table 3). Interestingly, aggresomes were found to have the most significant correlation with patient outcome in both OS (*P* = 0.01) and EFS (*P* = 0.02) as well as molecular subgroups; SHH OS (*P* = 0.06) and EFS (*P* = 0.03), WNT OS (*P* = 0.04) and EFS (*P* = 0.03), and non-WNT/non-SHH OS (*P* = 0.0002) and EFS (*P* = 0.0004). On the other hand, histological variants did not maintain their prognostic value (Table 3). Further evaluation of aggresomes’ percentage within the three risk groups showed that aggresomes’ percentage ≥20% identified patients with poor outcome only in SRMB OS (*P* = 0.02) and EFS (*P* = 0.06) (Fig. 2). However, there was no impact on both InfMB; OS (*P* = 1) and EFS (*P* = 0.3), and HRMB; OS (*P* = 0.1) and EFS (*P* = 0.2) patients (Supplementary Fig. 4).

**Table 2:** Univariate Cox regression analysis.

Variables	No. of cases	OS			EFS		
		HR	95% CI	*P-*value	HR	95% CI	*P*-value
**Histological subtypes**	88			0.007*			0.04*
Classic vs. D/N	49 vs. 12	0.3243	0.04–2.49	0.3	0.461	0.10–2	0.3
D/N vs. LC/A	12 vs. 27	0.1201	0.01–0.91	0.01*	0.236	0.05–1.04	0.04*
Classic vs. LC/A	49 vs. 27	2.583	1.19 -5.59	0.01*	2.018	0.98–4.14	0.05*
**Molecular subgroups**	88			0.08			0.05*
WNT vs. SHH	8 vs. 23	0.448	0.07–2.68	0.4	0.628	0.19–3.43	0.6
SHH vs. non WNT/non- SHH	23 vs. 57	0.2817	0.08–0.94	0.03*	0.306	0.10–0.87	0.02*
WNT vs. non-WNT/non-SHH	8 vs. 57	0.5612	0.13–2.39	0.4	0.466	0.11–1.96	0.3
**Age group**	88	4.133	0.56–30.4	0.1	2.472	0.59–10.3	0.2
**Stage**	82	1.16	0.51–2.62	0.7	1.124	0.53–2.36	0.8
**Residual tumor**	88	1.104	0.41–2.91	0.8	1.34	0.57–3.09	0.5
**Aggresomes’ percentage** **≥20%**	64 vs. 24	2.062	0.95–4.44	0.06	1.591	0.76–3.30	0.2

Estimated hazard ratio for OS and EFS with 95% confidence interval and P-value of the likelihood ratio test.

**Table 3:** Multivariate Cox proportional hazards analysis.

	OS			EFS		
	HR	95% CI	*P*-value	HR	95% CI	*P*-value
**Aggresomes’ percentage ≥20%**	3.419	1.30–8.93	0.01*	3.00	1.19–7.53	0.02*
**Histological subtypes**						
D/N	0.605	0.07–5.23	0.6	0.89	0.17–4.44	0.9
LC/A	1.91	0.78–4.63	0.2	1.64	0.72–3.73	0.2
**Molecular subgroups**						
SHH	0.273	0.06–1.07	0.06	0.25	0.07–0.89	0.03*
WNT	0.191	0.03–0.92	0.04*	0.17	0.03–0.84	0.03*
**Age** **g** **roup ≥3**	2.544	0.39–21.1	0.4	1.66	0.35–7.84	0.5

### Aggresomes increase the molecular stratification predictability of patients’ outcome

**Figure 3:**
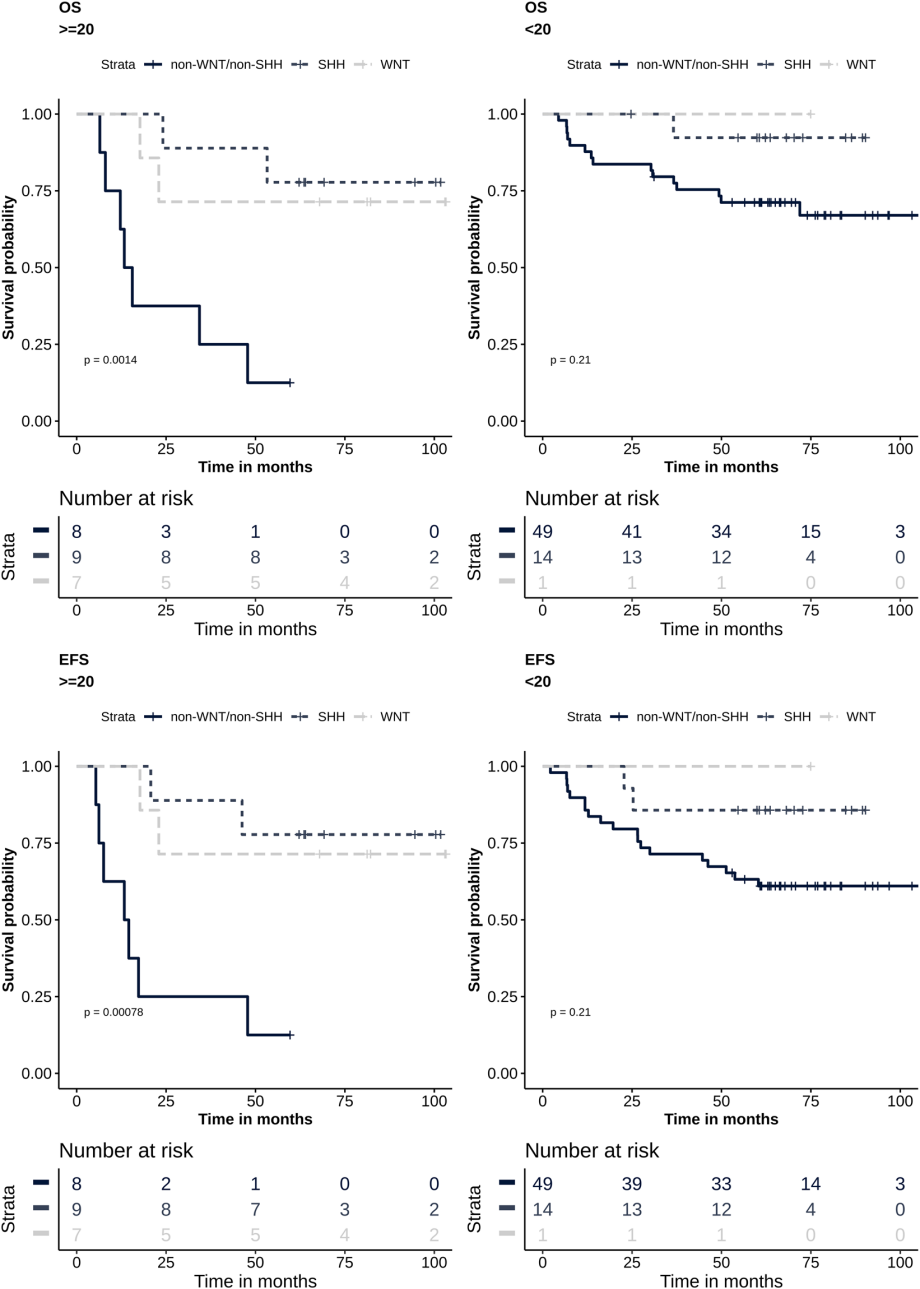
Aggresomes identify high-risk patients. Survival analysis Kaplan-Meier plot according to molecular signatures into WNT, SHH and non-WNT/non-SHH. Patients are stratified by percentage of aggresomes within the tumor into aggresome-positive (≥20%) and aggresomes-negative (<20%). Survival probability (y axis) and time indicated in months (x axis). *p-values* were calculated using the log-rank test.

Next, we determined the discriminatory power of aggresomes within molecular subgroups. Using C-index and time-dependent AUC, the 20% cut-off of aggresomes within molecular subgroups was found to improve outcome prediction compared to aggresomes independently (Supplementary Table 2). Furthermore, patients with aggresomes’ percentage ≥20% were significantly associated with poor outcome in non-WNT/non-SHH subgroup; OS (*P* = 0.0002) and EFS (*P* = 0.0004) (Fig. 2). However, molecular stratification was found to have a comparable discriminatory power (Supplementary Table 2). When stratifying patients first based on aggresomes’ percentage followed by molecular stratification, the predictive power of molecular marker increased in both OS (*P* = 0.001) and EFS (*P* = 0.0008) (Fig. 3, Supplementary Table 2). As for patients with aggresomes’ percentage <20%, the predictive value of molecular subgroups remained unaffected OS (*P* = 0.21) and EFS (*P* = 0.21) (Fig. 3, Supplementary Table 2).

## Discussion

In normal cells, aggresomes are transient juxta-nuclear structures which serve to eliminate defective aggregated proteins from accumulating in the cytoplasm^13,32^. Studies that investigate the underlying impact of aggresomes accumulation in cancer cell are rare. However, in multiple myeloma (MM), lymphoma and pancreatic cancer, aggresomes were detected following treatment with proteasome (Bortezomib) or deubiquitinase inhibitors (WP1130)^17,33^. These findings suggested that aggresomes formation is exploited by cancer cells to confer drug resistance and evade apoptotic signals caused by the impairment of the ubiquitin-proteasome axis^17,18,33,34^. Furthermore, we have previously reported the presence of aggresomes in CPC tumors^19^. Aggresomes were inherently part of the tumor prior to therapy, suggesting that their presence may provide a survival advantage to tumor cells^19^.

MB is the most common malignant brain tumor of childhood, with complex risk stratification that includes; histology, age at diagnosis, and size of residual tumor after surgical resection^26^. Advances in high throughput molecular profiling identified 4 distinct molecular signatures that guided clinical decision^27,29^. However, in a recent study, where over 700 pediatric MB patients were molecularly profiled, the authors identified 7 molecular subgroups^35^. While WNT remained the same, SHH, Group 3 and Group 4 were split into high-risk and low-risk groups, hence reflecting high degree of heterogeneity at the molecular level^35^. Such heterogeneity brings an additional challenge to our understanding of the biology of the disease and the impact on treatment options^35,36^. To determine if aggresomes are present in MB and examine their prognostic role, we screened 93 MB tumors retrospectively stratified into three molecular subgroups. Aggresomes were detected in all histological variants and were significantly associated with molecular subgroups. Furthermore, CCHE-188 cell line maintained aggresomes in 25% of the cells, similar to its parent tumor. This observation suggests that aggresomes are asymmetrically inherited. The exact reason for aggresome formation in MB is not yet known. However, their formation could be viewed as cellular response to maintain proteostasis. The colocalization of HDAC6 with vimentin only in aggresome-positive cells, supports the premise of the cytoprotective role of aggresomes by clearance of cytoplasmic misfolded/aggregated proteins and avoid proteotoxicity^37,38^.

Aggresomes’ percentage ranged from 1 to over 80% of tumor cells without prior knowledge to the cut-off value that should be used to determine its prognostic value. Therefore, we tested different cut-off values and found that the 20% cut-off provided the best discriminatory effect using AUC and C-index. On the contrary, the 30% and 60% cut-offs did not provide similar or higher AUC or C-index values. This may be attributed to lower number of patients in these two groups coupled with the molecular diversity of MB. Therefore, higher number of patients may be required to further test if the increase in aggresomes’ percentage may have a significant impact on prognosis. Upon examining all risk factors by univariate Cox proportional hazards analysis, histological variants were found to be the main contributor to both OS and EFS. Notably, molecular subgroups were only significant in EFS, while aggresomes’ percentage at ≥20% had a near significant impact on patients’ OS. When addressing the histology, molecular subtypes, age, and aggresomes’ percentage in multivariate analysis, aggresomes’ percentage ≥20% was the leading predictor of poor outcome. Moreover, WNT and SHH molecular subgroups predicted better patients’ outcome in OS and EFS. This affirmed previous studies where histology was not an adequate predictor of patients’ outcome, since each variant was comprised of different molecular entities. The importance of aggresomes in segregating patients with poor OS was also evident among standard risk patients. Since molecular subgroups are integrated into current patients risk stratification algorithm, we examined the effect of stratifying patients based on aggresomes’ percentage within each molecular subgroup. While patients with aggresomes’ percentage ≥20% had worse outcome in all molecular subgroups, only patients within the non-WNT/non-SHH subgroup had significantly lower OS and EFS. Stratifying patients into 2 groups according to aggresomes’ percentage within the tumor, followed by molecular stratification, increased the predictability of patients’ outcome. Moreover, molecular hierarchy was maintained where WNT and SHH subgroup patients had better outcome compared to non-WNT/non-SHH. On the other hand, there was no significant difference in patients’ outcome within the group with aggresomes’ percentage <20%. In addition to being an independent risk predictor, the inclusion of aggresomes as an initial screening step prior to molecular classification would significantly empower the prognostic value of the established molecular classification of MB patients.

While clinical based risk stratification was historically used to tailor treatment strategies, the integration of molecular signatures potentially provided a superior assessment to categorize patients and further improve treatment options. It must be noted that the molecular characterization techniques are very costly in terms of infrastructure and operation, making their integration into routine operation practically formidable, particularly in limited-resources and high patient-volume settings. This warrants the need for a simple, cost-effective prognostic tool. We believe that our results are encouraging to incorporate aggresomes assessment in the initial classification algorithm for MB, and is worth investigation in other studies with larger cohorts.

## Materials and Methods

### Patient cohort and tissue samples

The current retrospective study includes tumor samples from 93 archived formalin fixed paraffin embedded (FFPE) tissue blocks obtained from patients up to the age of 18 years. Samples were obtained from the Pathology Department at the Children’s Cancer Hospital Egypt 57357 (CCHE). Patient samples were collected for the period from 2009 to 2013 upon the approval of CCHE Institutional Review Board for Human Research (IRB). None of the patients had previously received chemotherapy or radiotherapy. Choice of tissue blocks was based on the presence of adequate tissue and largest tumor volume. Diagnosis was confirmed by two independent pathologists following World Health Organization (WHO) criteria of 2016^22^. Three histological subtypes were identified; classic, D/N, and LC/A. MB patients were assigned to receive one of the following three treatment protocols based on the Children Oncology Group (COG) treatment plans; the (COG-A9961) for SRMB patients, while HRMB patients were assigned to (COG-ACNS0332) protocol, and finally (COG – P9934) for InfMB patients.

### Multiplex ligation dependent probe amplification

20 µm sections were used to isolate DNA from FFPE tissue samples. DNA was then extracted using QIAamp DNA FFPE tissue kit (Qiagen, Valencia, CA, USA) according to the manufacturer’s instructions with the exception of deparaffinization using xylene which was repeated twice for 15 minutes each, and then washed twice using absolute ethanol^39^. DNA concentration was determined using Qubit 3.0 fluorometer (Invitrogen, Life Technologies, CA, USA) according to the manufacturer’s instructions. 150 ng of DNA were used to perform MLPA assay using ProFlex PCR System (Thermo-Fisher Scientific, Cambridge, UK) according to manufacturer’s protocol. SALSA MLPA KIT P301-A1 (MRC-Holland BV, Amsterdam, Netherlands) was used to identify amplifications and deletions in chromosomes 6, 14q, 16, and 17. Fragments were separated by capillary electrophoresis using the 3500 Genetic Analyzer (Applied Biosystems, CA, USA). Electrophoresis data was generated using Gene Mapper Software V5.0 (Applied Biosystems, CA, USA). Dosage quotient (DQ) was calculated using Coffalyser.Net Software (MRC Holland BV, Amsterdam, Netherlands) by comparing the relative probe peak in the tumor DNA to the reference DNA from normal kidney and brain FFPE tissue samples. DQ values < 0.8 and >1.2 were used as the cut-offs for heterozygous deletion and amplification, respectively, as recommended by manufacturer’s protocol. DQ values between the range of 0.8–1.2 were considered normal (normal = diploid).

### Establishment of medulloblastoma CCHE-188 cell line

MB cell line was generated from tumor sample with LC/A histology and non-WNT/non-SHH molecular subtype. The generation of the CCHE-188 cell line was performed as described previously^40^. The cell line establishment protocol was approved by the Children’s Cancer Hospital Egypt 57357 IRB. Fresh tumor sample obtained after surgery was cultured and maintained in RPMI 1640 (Lonza, Basel, Switzerland) supplemented with 10% calf bovine serum (Hyclone, GE Healthcare Life Sciences, Piscataway, NJ), 1% Pen-Strep (Gibco, Life Technologies, CA, USA) and maintained at 37 °C and 5% CO2. Short tandem repeats (STR) (Thermo-Fisher Scientific, Cambridge, UK) profiling was used to confirm cell line identity with respect to the original tumor.

### Immunohistochemistry and immunofluorescence analysis

IHC was performed on 4-μm FFPE tissue sections using Ventana Benchmark XT automated system (Ventana Medical System, Tucson, AZ, USA). For molecular stratification; WNT tumors were identified by positive nuclear staining of β-catenin (≥5%) and monosomy 6 identified by MLPA^27,28^, while SHH tumors were identified by positive cytoplasmic ± membranous immunostaining of GAB1(≥25%)^41^. For cases where GAB1 immunoreactivity was <25% of tumor tissue (n = 3), YAP1 was used to confirm SHH tumor classification. Cases negative for β-catenin and GAB1 or YAP1 were classified as non-WNT/non-SHH molecular subgroup. Aggresome cellular localization was detected using vimentin antibody. Slides were examined for the presence of a paranuclear round reaction in the cytoplasm. Accordingly, aggresome-positivity in MB tumor tissues were only considered for cells exhibiting a paranuclear dot of vimentin. Immunofluorescence (IF) was performed to confirm the presence of aggresomes in the established MB CCHE-188 cell line. Cells at passage 10–15 were seeded on coated coverslips with Poly D-Lysine (Merck Millipore, Germany) diluted 1:20 in PBS for 1 hour at room temperature. After 24 hours, cells were fixed with 4% formaldehyde (Sigma Aldrich, Chemie, Steinheim, Germany), permeabilized with 0.25% Triton-X-100 followed by blocking with 10% fetal bovine serum (Life Technologies, CA, USA) for 60 minutes. Cells were then incubated with rabbit anti-vimentin overnight. Cells were then washed 3 times with 1 X PBS, followed by incubation with secondary goat anti-rabbit antibody for 60 minutes at room temperature. Cells were counterstained with 4′, 6-diamidino-2-phenylindole (DAPI) (Abbot Molecular, Des Plains, IL, USA). Images were acquired using LSM 710 confocal scanning laser microscope (Carl Zeiss, Germany). Detailed information of antibodies used are listed in Supplementary Table 4.

### Statistical analysis

This retrospective study was designed without priori power analysis. Descriptive statistics were summarized for categorical variables as frequencies and percentages, and for continuous variables as the mean and the median. Statistical comparison of continuous factors was performed using Kruskal-Wallis test. Overall survival (OS) was defined as the time from diagnosis until death. Event-free survival (EFS) was defined as the time from diagnosis until disease progression, recurrence or death. OS and EFS estimation was performed using the Kaplan-Meier method. Differences between groups in OS or EFS were tested using log-rank tests. Univariate Cox proportional hazard regression analysis was performed for all risk factors. For the multivariate analysis, the following covariates were included; age, histology, molecular subgroups, and percent of aggresomes. For each variable, hazard ratio (HR) was calculated and its 95% confidence interval (CI) was determined. An effect was considered statistically significant if *p-value* was <0.05, AUC and C-index were calculated to determine the survival-based cut-off for aggresomes. All statistical analyses were performed using R statistical environment (v3.3.2) using survival^42^, survivalROC^43^, dynpred^44^, ComplexHeatmap^45^, and ggplot2^46^ packages. For the analysis of patients outcomes only 88 patients were included. Five patients were excluded; 3 patients underwent surgery without proceeding with treatment protocol and 2 patients were excluded as their risk stratification did not meet the inclusion criteria.

### Institutional review board statement

The study was performed in accordance with the declaration of Helsinki and experimental protocols was revised and approved by IRB at children’s Cancer Hospital Egypt. (22.10.2018)

### Informed consent statement

IRB at Children’s Cancer Hospital Egypt has approved the waiver of patient consent form because the study used archived pathological samples and analysis performed on the samples does not affect patient well-being in any way. Patient confidentiality is maintained at all time in accordance with Children’s Cancer Hospital Egypt policies.

## Supplementary information

Supplementary File

## Data Availability

All relevant data are available in the supplementary materials.
