# Supplementary material for: Association of Aggresomes with Survival Outcomes in Pediatric Medulloblastoma
Source: Sci Rep. 2019 Aug 30;9:12605. doi: 10.1038/s41598-019-49027-x (PMC6717208; doi:10.1038/s41598-019-49027-x)
Supplement: Supplementary file 1 — Supplementary File [file 41598_2019_49027_MOESM1_ESM.pdf]

Association of Aggresomes with Survival Outcomes in Pediatric Medulloblastoma

Authors

Maha Yehia [1, 15] * , Hala Taha [1, 9] *, Asmaa Salama [1, 9], Nada Amer [2], Amal Mosaab [2], Omneya Hassanain [3], Amal Refaat [4, 10], Dina Yassin [5, 11], Ahmed El-Hemaly [6, 12], Soha Ahmed [7, 13], Mohamed El-Beltagy [8, 14], Osama Shaalan [15] and Shahenda El-Naggar [2]

Affiliations

[1]Department of Pathology, Children's Cancer Hospital Egypt 57357, Cairo, Egypt, [2]Tumor Biology Research Program, Basic Research Unit, Department of Research, Children's Cancer Hospital Egypt 57357, Cairo, Egypt, [3]Clinical Research Unit, Department of Research, Children's Cancer Hospital Egypt 57357, Cairo, Egypt, [4]Department of Radiology, Children's Cancer Hospital Egypt 57357, Cairo, Egypt, [5]Laboratory of Molecular Biology, Department of Clinical Pathology, Children's Cancer Hospital Egypt 57357, Cairo, Egypt, [6]Department of Pediatric Oncology, Children's Cancer Hospital Egypt 57357, Cairo, Egypt, [7]Department of Radiotherapy, Children's Cancer Hospital Egypt 57357, Cairo, Egypt, [8]Department of Neurosurgery, Children's Cancer Hospital Egypt 57357, Cairo, Egypt, [9]Department of Pathology, National Cancer Institute, Cairo University, Cairo, Egypt, [10]Department of Radiology, National Cancer Institute, Cairo University, Cairo, Egypt, [11]Department of Clinical Pathology, National Cancer Institute, Cairo University, Cairo, Egypt, [12]Department of Pediatric Oncology, National Cancer Institute, Cairo University, Cairo, Egypt, [13]Department of Clinical Oncology, Faculty of Medicine, Aswan University, Aswan, Egypt, [14]Department of Neurosurgery, Faculty of Medicine, Cairo University, Cairo, Egypt, [15]Department of Molecular Diagnostics, Genetic Engineering and Biotechnology Research Institute, University of Sadat City, Menoufia, Egypt

* Maha Yehia and Hala Taha are co-first authors

Correspondence: Dr. Shahenda Mohamed El-Naggar, E-mail address: shahenda.elnaggar@57357.org

Research Department, Children's Cancer Hospital Egypt 57357, 1 Seket Al-Emam Street, Cairo, P.O box 11441, Egypt.

Supplementary Figure. 1

A

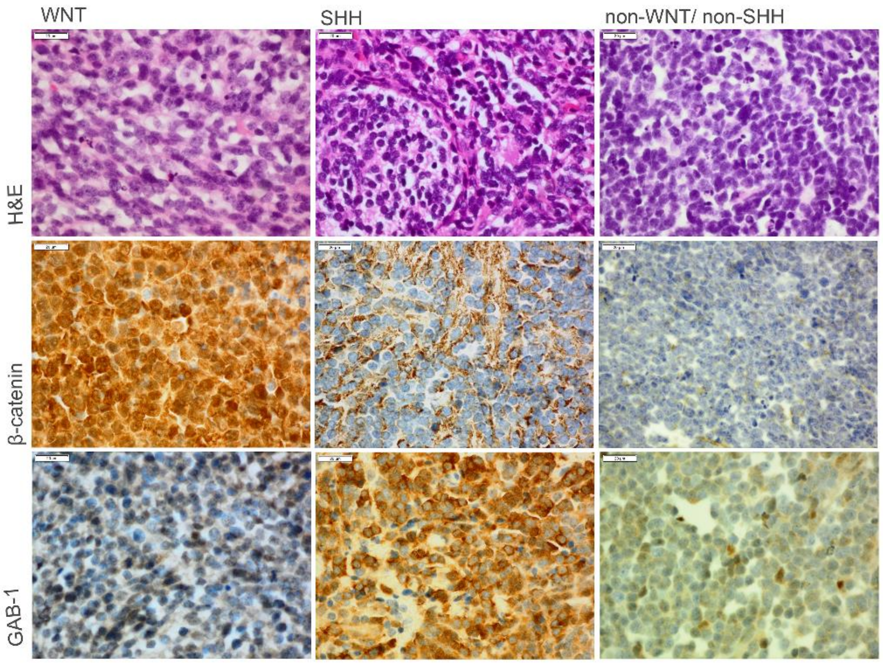

B

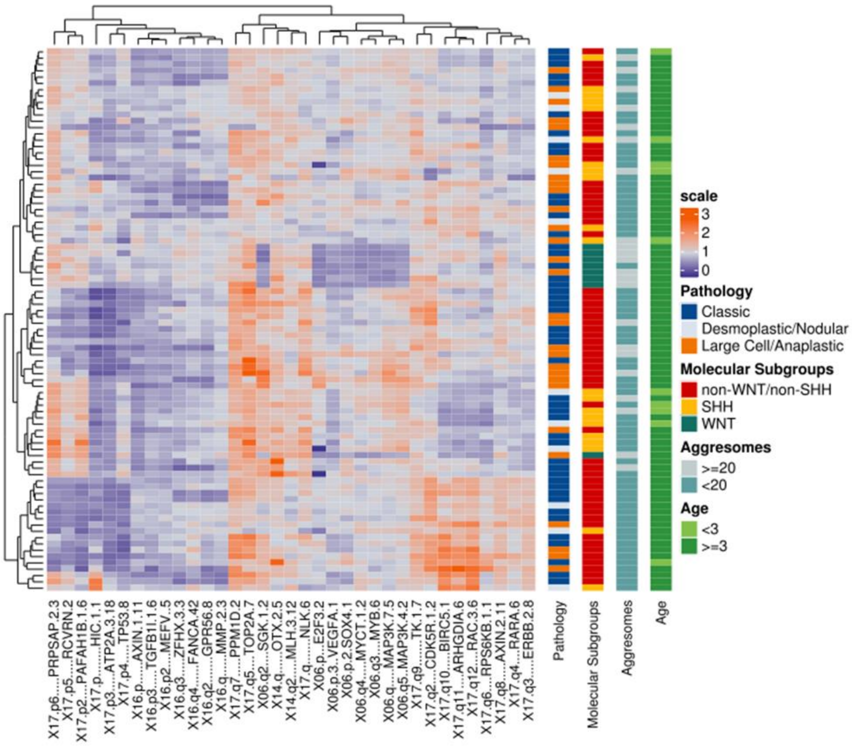

Supplementary Figure. 2

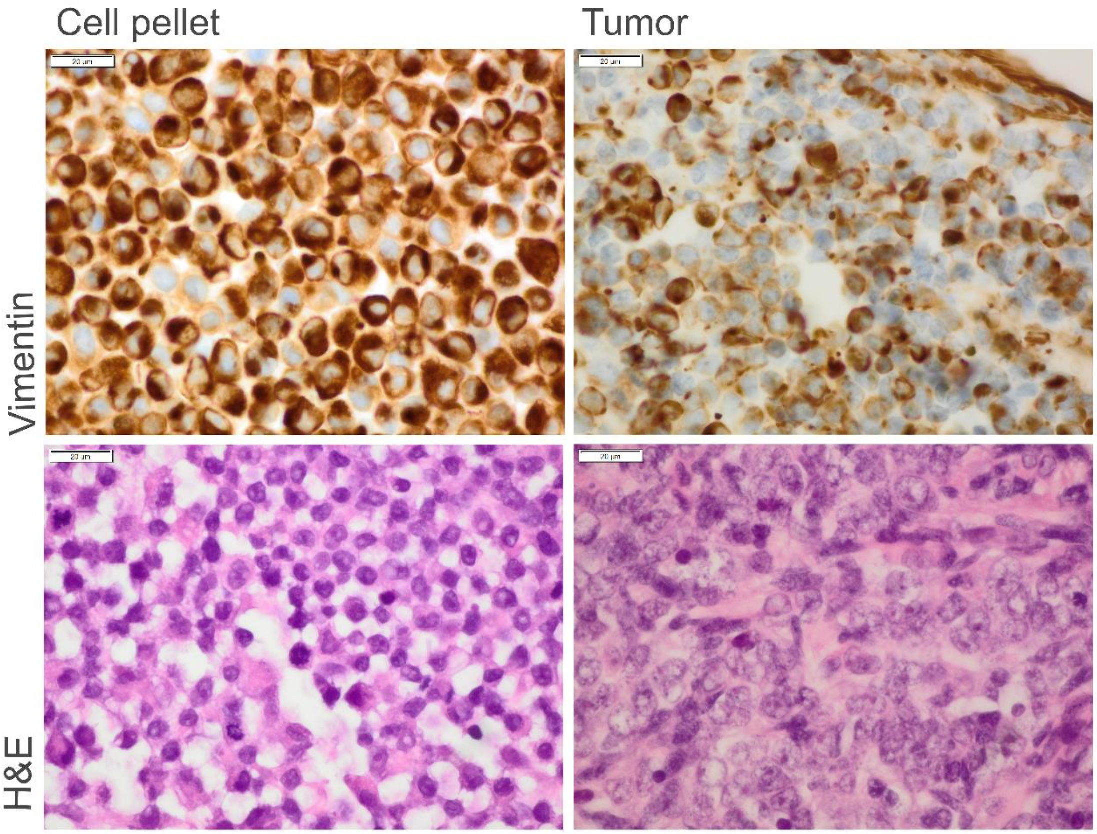

Supplementary Figure. 3

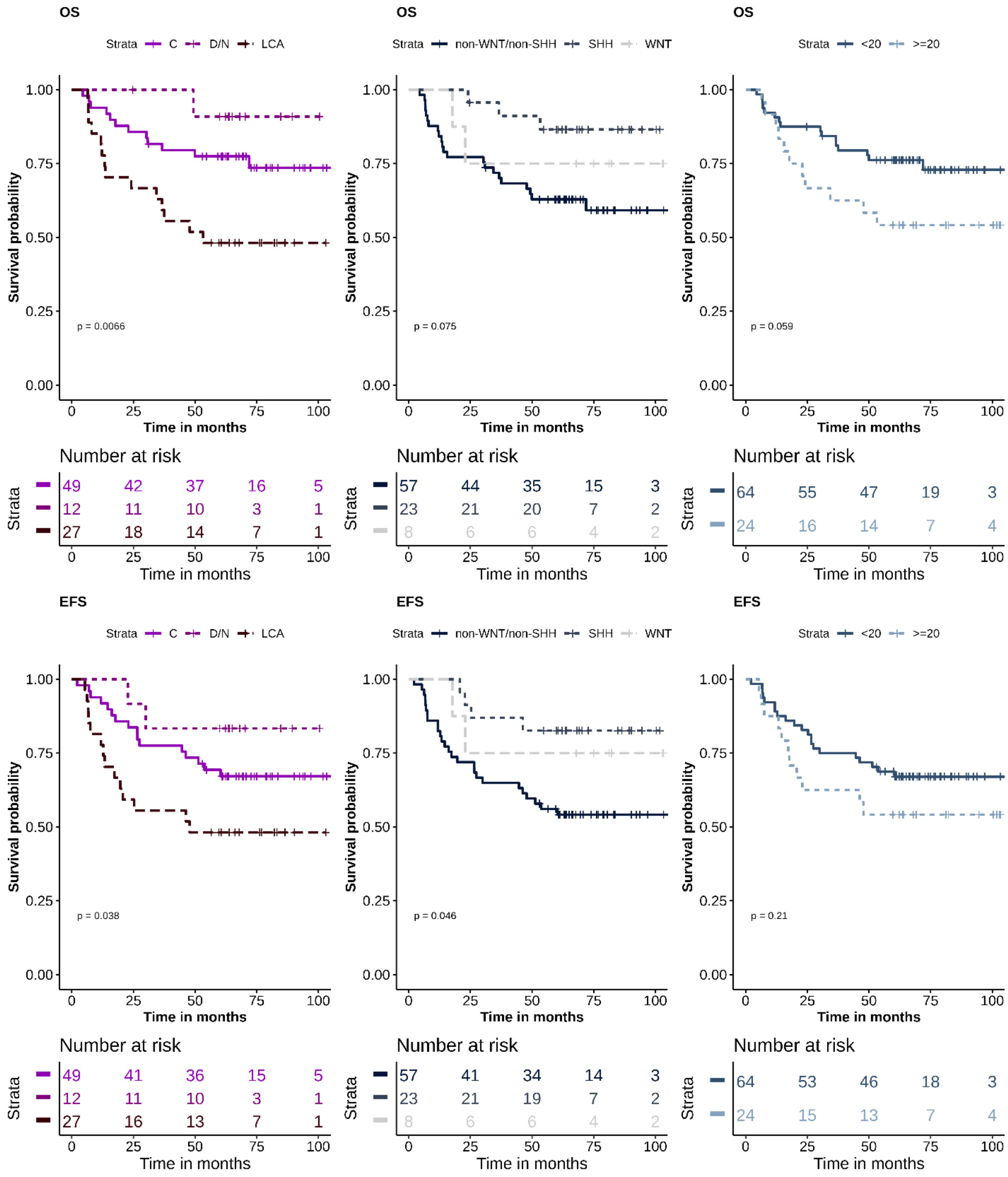

Supplementary Figure. 4

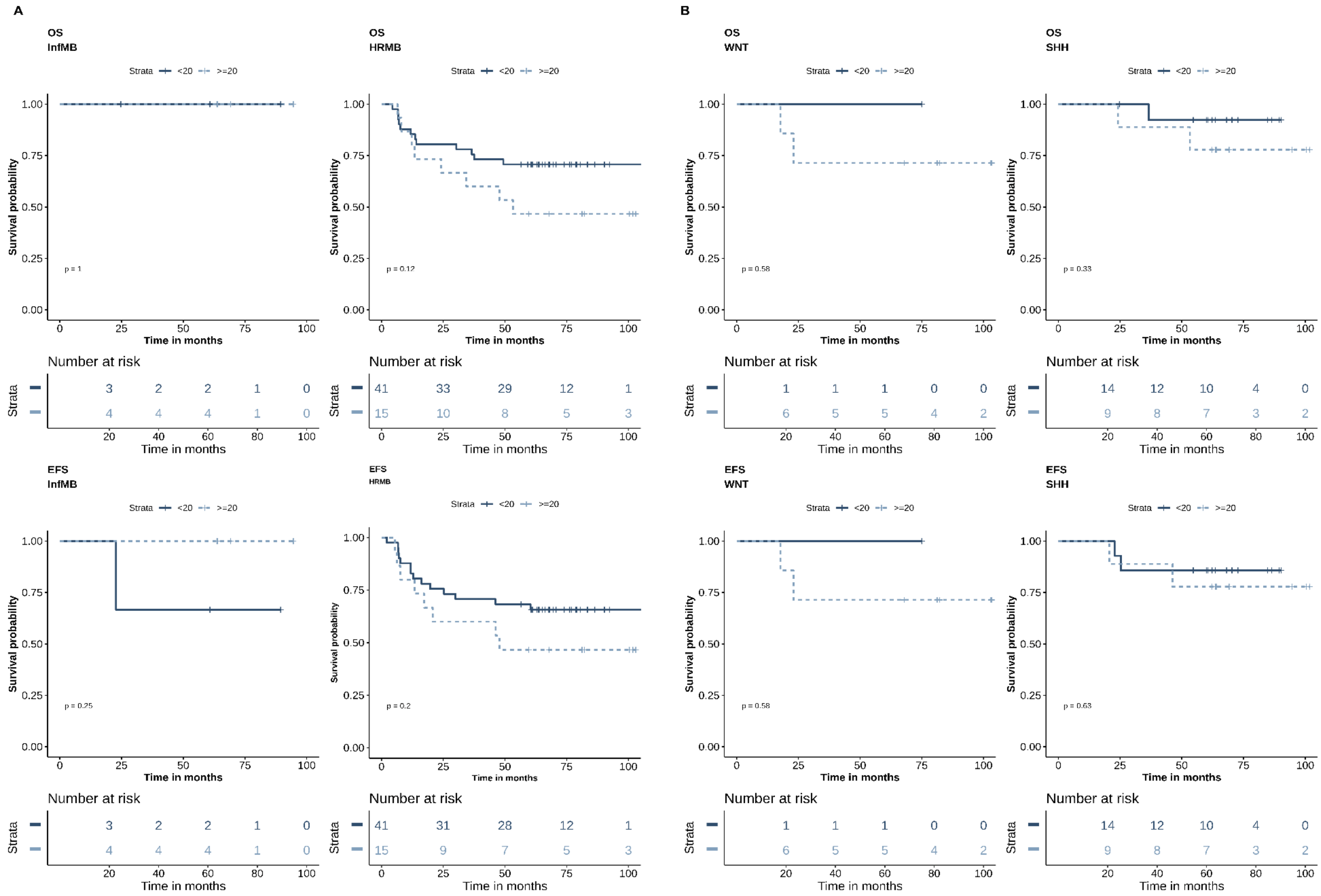

Supplementary Figure Legends

Supplementary Fig. 1. Stratification of medulloblastoma molecular subgroups. (A) Histologic evaluation was performed using H&E stained section. Nuclear β-catenin and cytoplasmic GAB-1 immunoreactivity were used as markers for WNT and SHH molecular subgroups respectively. Non-WNT/non-SHH subgroup were identified using cytoplasmic β-catenin and negative GAB-1 immunostaining; (B) Heatmap representation of MLPA DQ results. Clinicopathological and molecular annotations are provided as side bars according to the included key.

Supplementary Fig. 2. Immunohistochemical analysis of CCHE-188 cell pellet and the original tumor. Aggresomes are identified by the formation of juxta nuclear localization of vimentin in both cell line and tumor.

Supplementary Fig. 3. Survival analysis Kaplan-Meier plot of overall and event-free survival. Survival analysis separated by histological variants, molecular subgroups and percentage of aggresomes within the tumor (≥ 20% and < 20 %). Survival probability (y axis) and time indicated in months (x axis). *P-values* were calculated using the log-rank test.

Supplementary Fig. 4. Survival analysis Kaplan-Meier according to percentage of aggresomes within the tumor cells. (A) Patients are stratified according to clinicopathological risk criteria (InfMB and HRMB). (B) Patients are stratified using molecular subgrouping (WNT and SHH). Survival probability (y axis) and time indicated in months (x axis). *P-values* were calculated using the log-rank test.

Supplementary Table 1

ID	Gender	Pathology	β-catenin	GAB1	YAP1	M-subgroup	Vimentin	Aggresomes	M_Status	Treatment protocol
CCHE_Med_001	M	Classic	N	N	N/A	nonWnt/SHH	P	N	M0	HRMB (CCHE 3-1-2008)
CCHE_Med_002	M	Classic	N	N	N/A	nonWnt/SHH	N	N	M0	SRMB (CCHE 3-1-2008)
CCHE_Med_003	F	Desmoplastic/Nodular	N	90%	N/A	SHH	P	75%	M3	HRMB (CCHE 3-1-2008)
CCHE_Med_004	F	Classic	N	15%	N	nonWnt/SHH	N	N	M1	HRMB (CCHE 3-1-2008)
CCHE_Med_005	M	Large_Cell_Anaplastic	P	N	N/A	Wnt	P	20%	M0	HRMB (CCHE 3-1-2008)
CCHE_Med_006	F	Classic	P	N	N/A	Wnt	P	30%	M0	SRMB (CCHE 3-1-2008)
CCHE_Med_007	M	Classic	N	25%	N/A	SHH	P	85%	M0	HRMB (CCHE 3-1-2008)
CCHE_Med_008	M	Large_Cell_Anaplastic	N	N	N/A	nonWnt/SHH	N	N	Mx	Surgery only
CCHE_Med_009	M	Classic	N	N	N/A	nonWnt/SHH	N	N	M0	SRMB (CCHE 3-1-2008)
CCHE_Med_010	M	Large_Cell_Anaplastic	N	N	N/A	nonWnt/SHH	N	N	Mx	HRMB (CCHE 3-1-2008)
CCHE_Med_011	M	Classic	P	N	N/A	Wnt	P	60%	M0	SRMB (CCHE 3-1-2008)
CCHE_Med_012	F	Large_Cell_Anaplastic	N	N	N/A	nonWnt/SHH	P	20%	M0	HRMB (CCHE 3-1-2008)
CCHE_Med_014	M	Classic	N	N	N/A	nonWnt/SHH	N	N	M0	SRMB (CCHE 3-1-2008)
CCHE_Med_015	M	Classic	N	N	N/A	nonWnt/SHH	P	7%	M2	HRMB (CCHE 3-1-2008)
CCHE_Med_016	F	Classic	N	N	N/A	nonWnt/SHH	N	N	M0	SRMB (CCHE 3-1-2008)
CCHE_Med_017	M	Classic	N	N	N/A	nonWnt/SHH	N	N	M0	SRMB (CCHE 3-1-2008)
CCHE_Med_018	M	Large_Cell_Anaplastic	P	N	P	Wnt	P	60%	M0	HRMB (CCHE 3-1-2008)
CCHE_Med_019	M	Classic	N	N	N/A	nonWnt/SHH	N	N	M1	HRMB (CCHE 3-1-2008)
CCHE_Med_020	F	Classic	P	N	N/A	Wnt	P	70%	M1	HRMB (CCHE 3-1-2008)
CCHE_Med_021	F	Large_Cell_Anaplastic	N	N	N/A	nonWnt/SHH	N	N	M1	HRMB (CCHE 3-1-2008)
CCHE_Med_022	F	Large_Cell_Anaplastic	N	98%	N/A	SHH	N	15%	Mx	HRMB (CCHE 3-1-2008)
CCHE_Med_023	F	Large_Cell_Anaplastic	N	90%	P	SHH	P	2%	M0	HRMB (CCHE 3-1-2008)
CCHE_Med_024	M	Classic	N	N	N/A	nonWnt/SHH	N	N	M1	HRMB (CCHE 3-1-2008)
CCHE_Med_025	F	Classic	N	75%	N/A	SHH	P	90%	M0	InfMB (CCHE 3-1-2008)
CCHE_Med_026	M	Desmoplastic/Nodular	N	40%	N/A	SHH	N	5%	M0	SRMB (CCHE 3-1-2008)
CCHE_Med_027	M	Classic	N	N	N/A	nonWnt/SHH	N	N	Mx	HRMB (CCHE 3-1-2008)
CCHE_Med_028	M	Large_Cell_Anaplastic	N	N	N/A	nonWnt/SHH	N	10%	M1	HRMB (CCHE 3-1-2008)
CCHE_Med_029	M	Desmoplastic/Nodular	N	50%	N/A	SHH	P	15%	Mx	InfMB (CCHE 3-1-2008)
CCHE_Med_030	M	Classic	N	N	N/A	nonWnt/SHH	N	N	M1	HRMB (CCHE 3-1-2008)
CCHE_Med_031	F	Classic	P	N	N/A	Wnt	P	20%	M0	SRMB (CCHE 3-1-2008)
CCHE_Med_032	M	Classic	N	N	N/A	nonWnt/SHH	P	5%	M0	SRMB (CCHE 3-1-2008)
CCHE_Med_033	M	Large_Cell_Anaplastic	N	40%	N/A	SHH	N	10%	Mx	HRMB (CCHE 3-1-2008)

CCHE_Med_034	F	Desmoplastic/Nodular	N	N	P	SHH	N	1%	M0	InfMB (CCHE 3-1-2008)
CCHE_Med_035	M	Large_Cell_Anaplastic	N	N	N/A	nonWnt/SHH	P	2%	M1	HRMB (CCHE 3-1-2008)
CCHE_Med_036	M	Large_Cell_Anaplastic	N	N	N/A	nonWnt/SHH	P	5%	M0	SRMB (CCHE 3-1-2008)
CCHE_Med_038	M	Classic	N	N	N/A	nonWnt/SHH	N	N	M0	SRMB (CCHE 3-1-2008)
CCHE_Med_039	M	Classic	N	N	N/A	nonWnt/SHH	P	7%	M0	HRMB (CCHE 3-1-2008)
CCHE_Med_040	M	Classic	N	N	N/A	nonWnt/SHH	P	25%	M0	SRMB (CCHE 3-1-2008)
CCHE_Med_041	F	Large_Cell_Anaplastic	N	N	N/A	nonWnt/SHH	P	15%	M1	HRMB (CCHE 3-1-2008)
CCHE_Med_042	F	Desmoplastic/Nodular	N	98%	N/A	SHH	N	N	M0	SRMB (CCHE 3-1-2008)
CCHE_Med_045	F	Classic	N	N	N/A	nonWnt/SHH	N	N	M0	SRMB (CCHE 3-1-2008)
CCHE_Med_046	F	Large_Cell_Anaplastic	N	N	N/A	nonWnt/SHH	N	N	M1	HRMB (CCHE 3-1-2008)
CCHE_Med_047	M	Classic	N	N	N/A	nonWnt/SHH	P	5%	M1	HRMB (CCHE 3-1-2008)
CCHE_Med_048	M	Classic	N	2%	N	nonWnt/SHH	N	2%	M2	HRMB (CCHE 3-1-2008)
CCHE_Med_049	M	Large_Cell_Anaplastic	N	N	N/A	nonWnt/SHH	N	1%	M1	HRMB (CCHE 3-1-2008)
CCHE_Med_050	M	Large_Cell_Anaplastic	N	N	N/A	nonWnt/SHH	N	2%	M3	HRMB (CCHE 3-1-2008)
CCHE_Med_051	M	Classic	N	90%	N/A	SHH	N	10%	M0	SRMB (CCHE 3-1-2008)
CCHE_Med_052	F	Classic	N	N	N/A	nonWnt/SHH	N	1%	M0	HRMB (CCHE 3-1-2008)
CCHE_Med_053	M	Classic	P	N	N/A	Wnt	P	10%	M0	SRMB (CCHE 3-1-2008)
CCHE_Med_054	M	Classic	N	N	N/A	nonWnt/SHH	N	1%	M1	HRMB (CCHE 3-1-2008)
CCHE_Med_055	M	Large_Cell_Anaplastic	N	N	N/A	nonWnt/SHH	P	20%	M0	HRMB (CCHE 3-1-2008)
CCHE_Med_056	M	Classic	N	N	N/A	nonWnt/SHH	N	N	M3	Surgery only
CCHE_Med_057	F	Classic	N	25%	N/A	SHH	P	N	M0	SRMB (CCHE 3-1-2008)
CCHE_Med_058	F	Large_Cell_Anaplastic	N	80%	N/A	SHH	P	80%	M0	HRMB (CCHE 3-1-2008)
CCHE_Med_059	M	Classic	N	N	N/A	nonWnt/SHH	N	N	M0	SRMB (CCHE 3-1-2008)
CCHE_Med_060	F	Large_Cell_Anaplastic	N	N	N/A	nonWnt/SHH	N	2%	M0	HRMB (CCHE 3-1-2008)
CCHE_Med_061	M	Large_Cell_Anaplastic	N	N	N/A	nonWnt/SHH	N	N	M0	HRMB (CCHE 3-1-2008)
CCHE_Med_062	F	Large_Cell_Anaplastic	N	85%	N/A	SHH	P	80%	M0	HRMB (CCHE 3-1-2008)
CCHE_Med_063	F	Large_Cell_Anaplastic	N	N	N/A	nonWnt/SHH	P	20%	M3	HRMB (CCHE 3-1-2008)
CCHE_Med_065	F	Desmoplastic/Nodular	N	25%	N/A	SHH	N	N	M0	SRMB (CCHE 3-1-2008)
CCHE_Med_066	M	Classic	N	N	N/A	nonWnt/SHH	N	N	M3	HRMB (CCHE 3-1-2008)
CCHE_Med_068	F	Classic	N	N	N/A	nonWnt/SHH	N	N	M2	HRMB (CCHE 3-1-2008)
CCHE_Med_069	F	Classic	N	N	N/A	nonWnt/SHH	N	5%	M0	HRMB (CCHE 3-1-2008)
CCHE_Med_070	F	Large_Cell_Anaplastic	N	N	N/A	nonWnt/SHH	P	70%	M0	HRMB (CCHE 3-1-2008)
CCHE_Med_071	M	Classic	N	N	N/A	nonWnt/SHH	N	5%	M0	HRMB (CCHE 3-1-2008)
CCHE_Med_072	M	Desmoplastic/Nodular	N	N	FWN	nonWnt/SHH	P	1%	M3	HRMB (CCHE 3-1-2008)
CCHE_Med_073	M	Classic	N	N	N/A	nonWnt/SHH	P	3%	M3	HRMB (CCHE 3-1-2008)

CCHE_Med_074	M	Large_Cell_Anaplastic	N	N	N/A	nonWnt/SHH	N	N	M1	Surgery only
CCHE_Med_075	F	Desmoplastic/Nodular	N	40%	N/A	SHH	P	60%	M1	InfMB (CCHE 3-1-2008)
CCHE_Med_077	F	Classic	N	90%	N/A	SHH	P	80%	M0	SRMB (CCHE 3-1-2008)
CCHE_Med_078	M	Large_Cell_Anaplastic	N	N	N/A	nonWnt/SHH	P	90%	M3	HRMB (CCHE 3-1-2008)
CCHE_Med_079	F	Classic	N	N	N/A	nonWnt/SHH	N	N	M0	HRMB (CCHE 3-1-2008)
CCHE_Med_080	M	Large_Cell_Anaplastic	N	N	N/A	nonWnt/SHH	P	40%	M0	SRMB (CCHE 3-1-2008)
CCHE_Med_081	F	Classic	N	7%	P	SHH	P	40%	M1	InfMB (CCHE 3-1-2008)
CCHE_Med_082	F	Desmoplastic/Nodular	N	45%	N/A	SHH	P	35%	Mx	InfMB (CCHE 3-1-2008)
CCHE_Med_083	F	Desmoplastic/Nodular	N	98%	N/A	SHH	N	N	M0	HRMB (CCHE 3-1-2008)
CCHE_Med_084	F	Classic	N	N	N/A	nonWnt/SHH	P	N	M0	HRMB (CCHE 3-1-2008)
CCHE_Med_085	F	Classic	N	N	N/A	nonWnt/SHH	N	1%	M0	InfMB (CCHE 3-1-2008)
CCHE_Med_086	M	Large_Cell_Anaplastic	N	N	N/A	nonWnt/SHH	P	20%	M0	HRMB (CCHE 3-1-2008)
CCHE_Med_087	M	Desmoplastic/Nodular	N	N	FWN	nonWnt/SHH	N	8%	M1	HRMB (CCHE 3-1-2008)
CCHE_Med_088	F	Classic	N	N	N/A	nonWnt/SHH	N	N	M1	HRMB (CCHE 3-1-2008)
CCHE_Med_089	F	Classic	N	N	N/A	nonWnt/SHH	P	5%	M0	SRMB (CCHE 3-1-2008)
CCHE_Med_090	M	Classic	N	N	N/A	nonWnt/SHH	N	N	M3	HRMB (CCHE 3-1-2008)
CCHE_Med_091	M	Large_Cell_Anaplastic	P	N	N/A	Wnt	P	20%	M0	HRMB (CCHE 3-1-2008)
CCHE_Med_092	M	Classic	N	N	N/A	nonWnt/SHH	N	N	M3	HRMB (CCHE 3-1-2008)
CCHE_Med_093	F	Classic	N	N	N/A	nonWnt/SHH	P	10%	M0	SRMB (CCHE 3-1-2008)
CCHE_Med_094	F	Large_Cell_Anaplastic	N	90%	N/A	SHH	P	10%	M0	HRMB (CCHE 3-1-2008)
CCHE_Med_095	F	Large_Cell_Anaplastic	N	N	N/A	nonWnt/SHH	P	10%	M1	HRMB (CCHE 3-1-2008)
CCHE_Med_096	F	Large_Cell_Anaplastic	N	N	N	nonWnt/SHH	P	55%	M1	HRMB (CCHE 3-1-2008)
CCHE_Med_097	F	Classic	N	N	N/A	nonWnt/SHH	N	N	M3	HRMB (CCHE 3-1-2008)
CCHE_Med_098	M	Classic	N	N	N/A	nonWnt/SHH	N	N	M0	SRMB (CCHE 3-1-2008)
CCHE_Med_099	M	Desmoplastic/Nodular	N	60%	N/A	SHH	N	2%	M0	SRMB (CCHE 3-1-2008)
CCHE_Med_100	M	Classic	N	85%	N/A	SHH	P	5%	M0	SRMB (CCHE 3-1-2008)

FWN= Focal weak nuclear

Supplementary Table 2

	OS		EFS	
	**C-Index**	**AUC**	**C-Index**	**AUC**
5 % Aggresomes	0.5	0.51	0.53	0.51
10 % Aggresomes	0.53	0.54	0.52	0.54
20 % Aggresomes	0.58	0.59	0.55	0.59
30 % Aggresomes	0.5	0.5	0.52	0.52
60 % Aggresomes	0.52	0.53	0.5	0.51
MS	0.62	0.62	0.62	0.62
WNT	0.58	0.51	0.58	0.51
SHH	0.65	0.65	0.56	0.57
non-WNT/non-SHH	0.62	0.64	0.60	0.62
≥ 20% Aggresomes + MS	0.76	0.79	0.77	0.79
< 20 Aggresomes + MS	0.60	0.62	0.59	0.60

Supplementary Table 3

Antibody	Manufacturer (catalog no.)	Application	Dilution	Localization	Target
β-catenin	Dako (IS702)	IHC	1:100	Nuclear	WNT subgroup
GAB1	GeneTex (GTX111253)	IHC	1:100	Cytoplasmic ± membranous	SHH subgroup
YAP1	Santa Cruz Biotechnology (G-6; sc-376830)	IHC	1:100	Cytoplasmic	Confirm WNT, SHH subgroup
Vimentin	Ventana (790-2917)	IHC	1:100	Cytoplasmic	Aggresomes
Vimentin	Abcam (ab8978)	IF	1:50	Cytoplasmic	Aggresomes
Secondary goat anti-rabbit	Cell Signaling Technology (Alexa Fluor 488)	IF	1:500		
HDAC6	Cell Signaling Technology (7558)	IF	1:200	Cytoplasmic	HDAC6
Secondary goat anti-mouse	Cell Signaling Technology (AlexaFluor 555)	IF	1:500		

HDAC6 = histone deacetylase 6, IHC= Immunohistochemistry, IF= Immunofluorescence
